# The Role of Imaging in Measuring Disease Progression and Assessing Novel Therapies in Aortic Stenosis

**DOI:** 10.1016/j.jcmg.2018.10.023

**Published:** 2019-01

**Authors:** Mhairi K. Doris, Russell J. Everett, Matthew Shun-Shin, Marie-Annick Clavel, Marc R. Dweck

**Affiliations:** a British Heart Foundation Centre for Cardiovascular Science, University of Edinburgh, Edinburgh, Scotland, United Kingdom; b National Heart and Lung Institute, Imperial College London, London, United Kingdom; c Department of Medicine, Québec Heart and Lung Institute, Laval University, Québec City, Québec, Canada

**Keywords:** aortic stenosis, disease progression, noninvasive imaging, novel therapies, AS, aortic stenosis, AU, arbitrary unit(s), AVA, aortic valve area, CMR, cardiac magnetic resonance, CT, computed tomography, CT-AVC, computed tomography aortic valve calcification, ECV, extracellular volume, ^18^F-NaF, radiolabeled sodium fluoride, ICC, intraclass correlation coefficient, LGE, late gadolinium enhancement, Lp(a), lipoprotein A, LV, left ventricle, MGP, matrix Gla protein, PET, positron emission tomography, RANKL, receptor activator of nuclear factor kappa-B ligand

## Abstract

Aortic stenosis represents a growing health care burden in high-income countries. Currently, the only definitive treatment is surgical or transcatheter valve intervention at the end stages of disease. As the understanding of the underlying pathophysiology evolves, many promising therapies are being investigated. These seek to both slow disease progression in the valve and delay the transition from hypertrophy to heart failure in the myocardium, with the ultimate aim of avoiding the need for valve replacement in the elderly patients afflicted by this condition. Noninvasive imaging has played a pivotal role in enhancing our understanding of the complex pathophysiology underlying aortic stenosis, as well as disease progression in both the valve and myocardium. In this review, the authors discuss the means by which contemporary imaging may be used to assess disease progression and how these approaches may be utilized, both in clinical practice and research trials exploring the clinical efficacy of novel therapies.

## Central Illustration

**Figure:**
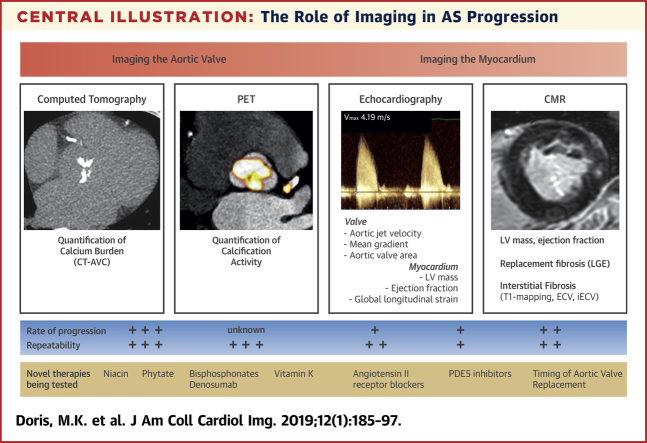

Aortic stenosis (AS) affects over 7 million people over 75 years of age in Europe and North America, and its prevalence is expected to triple in the next 40 years 1, 2, 3. The development of effective medical therapy is a major unmet clinical need that will require both a greater understanding of the underlying pathophysiology and the adoption of novel imaging methods to establish the safety and efficacy of candidate drugs. AS is a fibrocalcific disease in which deposition of lipid, collagen, and calcification leads to thickening and immobility of the aortic valve leaflets, resulting in progressive valve narrowing and obstruction to left ventricular outflow. Over time, the left ventricle (LV) responds to the consequent increase in afterload by myocyte hypertrophy, extracellular expansion, and ultimately myocardial fibrosis and decompensation 4, 5. In this review, we will discuss how these complex pathophysiological processes might be monitored with modern imaging techniques and ultimately modulated using novel therapeutic interventions.

## Pathophysiology of AS

### The valve

The initiation phase of AS shares many pathophysiological similarities with atherosclerosis and is dominated by inflammation, lipid infiltration, and extracellular matrix remodeling under the control of inflammatory signaling pathways 6, 7, 8, 9, 10. Like atherosclerosis, the initiating insult appears to be a combination of increased mechanical and oxidative stress or reduced shear stress leading to endothelial damage and a powerful inflammatory response. As this process continues, inflammatory signaling pathways are superseded by a powerful and relentless cycle of progressive calcification, coordinated by osteoblast-like cells and governed by pro-osteogenic signaling pathways 4, 11, 12. The accumulation of calcium within the valve during the later propagation phase induces further injury, thereby establishing a vicious cycle of accelerating calcification and progressive valvular obstruction.

### The myocardium

In addition to progressive valvular obstruction, AS has direct effects on the LV. Progressive valve obstruction results in an increased afterload, triggering myocyte hypertrophy and compensatory wall thickening that initially preserves wall stress and maintains cardiac output. Over time, however, cellular hypertrophy progresses to myocyte death, expansion of the extracellular space, and replacement fibrosis 5, 13, 14, 15, 16. Indeed myocyte death and myocardial fibrosis drive LV decompensation and the transition from hypertrophy to heart failure.

## What Do We Require of Imaging in AS?

Cardiac imaging is pivotal to the management of AS and is relied on to confirm the diagnosis and to grade stenosis severity and assess myocardial health both at baseline and over time. AS is a slowly progressive condition that advances at a variable and inconsistent rate. To accurately measure or predict disease progression over time, imaging tests must have sufficient reproducibility and robustness to detect small changes in disease severity with high accuracy. In the clinical setting, this is of utmost importance when selecting the appropriate management strategy and optimal timing of intervention for the individual patient. In the research setting, these attributes are crucial in clinical trials seeking to investigate efficacy of novel therapies. Indeed, imaging biomarkers with improved repeatability and sensitivity to change will minimize sample sizes, follow-up duration, and the expense of trials. Drug trials using imaging endpoints therefore desire biomarkers that maximize the progression-to-noise ratio: the ratio between the magnitudes of average progression in a particular parameter compared with its scan-rescan repeatability (the error in measuring that parameter on 2 different scans). Although transthoracic echocardiography has remained the gold standard method for assessing the aortic valve and myocardium and the tool of choice in the clinical setting, novel imaging techniques demonstrate potential advantages and are therefore being increasingly explored, particularly in clinical trials of novel therapies (Central Illustration).

**Central Illustration:**
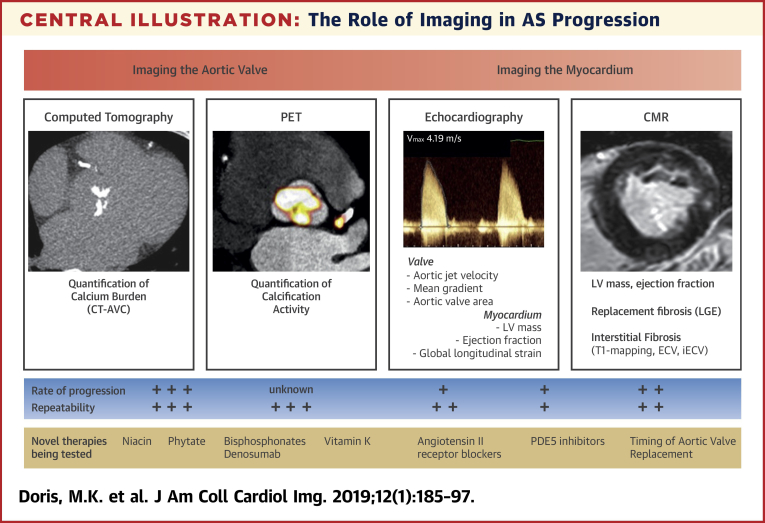
The Role of Imaging in AS Progression Noninvasive imaging provides the ability to directly image the aortic valve and consequent pathophysiological effects on the myocardium. Imaging the valve may be performed by computed tomography (CT) to quantify aortic valve calcification (AVC) load (CT-AVC), positron emission tomography (PET) to measure calcification activity and/or echocardiography to assess hemodynamic severity. The response of the left ventricle (LV) may be assessed by echocardiographic measures of mass, ejection fraction and/or strain, whereas cardiac magnetic resonance (CMR) offers additional quantification of fibrosis. The ability of these techniques to detect therapeutic efficacy depends on the scan-rescan repeatability of the test and the rate of change of the parameter being measured. These attributes are being exploited in a number of ongoing studies to investigate novel therapies for aortic stenosis. AS = aortic stenosis; ECV = extracellular volume; iECV = indexed extracellular volume; LGE = late gadolinium enhancement; PDE5 = phosphodiesterase type 5.

## Assessing the Valve

### Echocardiography

While there may not be a true reference standard for AS severity, echocardiography is considered the gold standard for clinical assessment. Early work in the 1970s found that Doppler ultrasound could be used to examine the jets of stenotic and regurgitant lesions (17). This laid the groundwork for Hatle et al. 18, 19 to demonstrate that Doppler ultrasound was highly feasible in AS and the peak jet velocity, converted into a gradient using the simplified Bernoulli equation (20), had good agreement with invasive measurements. Otto et al. (21), along with many other groups, have since demonstrated that echocardiographic biomarkers strongly predict the need for intervention. Finally, the use of echocardiographic markers of severity as enrolment criteria in the PARTNER B (Placement of Aortic Transcatheter Valve) trial (22) demonstrates the ability of echocardiography to select patients for beneficial therapy. Combined with the absence of radiation, widespread availability, and low imaging costs, these characteristics place echocardiography as the first-line modality for screening and serial follow-up in AS.

Although multiple echocardiographic parameters exist to assess disease severity, current guidelines recommend the assessment of severity and progression based on peak velocity, mean gradient, and aortic valve area (AVA) (23). Each of these central parameters have been found to predict outcome across multiple studies 24, 25, 26. Aortic peak velocity remains the first-line biomarker in the European Society of Cardiology/European Association for Cardio-Thoracic Surgery 2017 guidelines, providing powerful prognostic information and superior reproducibility than other parameters 27, 28. However, peak velocity is dependent on flow status and accurate alignment of the echocardiography probe with the jet of blood through the valve. Whereas mean gradient is subject to the same limitations as peak velocity, AVA is in principle less flow-dependent. However, measurements of AVA can represent an important source of discrepancy, particularly as a result of variations in direct measurements of the LV outflow tract. As LV outflow tract diameter is squared to provide AVA by the continuity equation, small differences in measurement can lead to significant variation and often to an underestimation of AVA. Echocardiographic measurements therefore display considerable variability, potentially leading to inaccuracies when estimating disease progression. Moreover, echocardiographic measurements demonstrate relatively slow progression with time. As a consequence, the progression-to-noise ratio for most echocardiographic assessments is not favorable 29, 30. Although this is often not a major issue in clinical practice, in the research setting it means that clinical trials require relatively large numbers of patients and prolonged follow-up to detect true treatment effects (29).

An additional limitation worth considering is that echocardiographic measures of AS often provide conflicting assessments of disease severity. Indeed, discordant echocardiographic results are seen in one-quarter of patients, most often arising from a valve area <1 cm^2^, suggesting severe disease, and a peak velocity <4.0 m/s or mean gradient <40 mm Hg, indicating moderate stenosis 31, 32. In cases of low-flow, low-gradient AS, flow can be temporarily increased to assess the true hemodynamic severity of aortic valve disease. Low-dose dobutamine stress echocardiography is often useful in this regard—with an increase in velocity with increased flow rates used to diagnose true severe AS (33) and to discriminate patients with and without contractile reserve (34). However, a significant proportion of patients with discordant findings are in fact found to have normal flow status, making echocardiographic results difficult to interpret.

For these reasons, interest in developing novel assessments of disease severity and progression in AS is growing, and novel imaging techniques may complement echocardiography in adjudicating disease severity and monitoring disease progression.

## Computed Tomography Calcium Scoring

**Figure 1:**
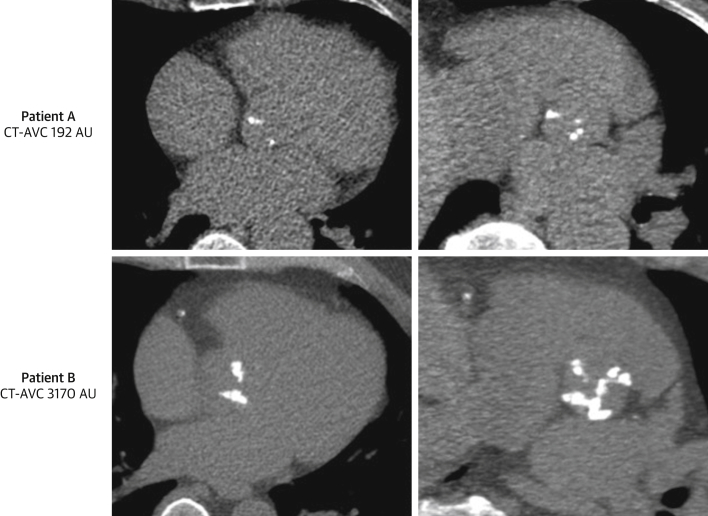
CT Calcium Scoring of the Aortic Valve An example of mild aortic valve calcification (AVC) (Patient A, **top**) and severe calcification by computed tomography (CT) (Patient B, **bottom**) in axial **(left)** and short-axis **(right)** views of the valve. AU = arbitrary unit(s).

Calcification is the predominant process causing valve obstruction in AS. Quantification of the calcium burden has therefore been suggested as an alternative flow-independent method of determining disease severity (35). This was first demonstrated using a semiquantitative assessment on echocardiography 35, 36, although the clinical utility of this approach has been limited by subjectivity and suboptimal reproducibility (37). Interest has instead turned to computed tomography (CT) calcium scoring, an established clinical technique already widely used to quantify coronary arterial calcification. Using a noncontrast, electrocardiography-gated CT acquisition and similar protocols as for coronary calcium scoring, the burden of valvular calcium can be quantified using the Agatston method, which encompasses both the area and weighted density of a given region of calcification (Figure 1).

Early studies of computed tomography aortic valve calcification (CT-AVC) scoring highlighted that this could provide a complementary measure of stenosis severity, correlating moderately with hemodynamic severity on echocardiography 37, 38. A major advance was the realization that men and women require different calcium scores to develop severe stenosis, even when adjusted for body size or LV outflow dimensions, and therefore that sex-specific thresholds were needed to grade severity (39). In 646 patients with normal LV systolic function, the application of calcium score thresholds of 2,065 (arbitrary units [AU]) for men and 1,274 (AU) for women correctly classified severe AS with a sensitivity of ≥86% and specificity of ≥79% (31). These thresholds have been validated in a further multicenter cohort of over 900 patients, holding particular potential in adjudicating disease severity in patients with discordant echocardiography results (32).

In addition to defining severity, CT-AVC offers powerful prediction of future clinical events. Indeed in recent multicenter studies, severe calcification was associated with a 3- to 4-fold increase in death or AVR 32, 40, emerging as an independent predictor of these events after adjustment for clinical and echocardiographic parameters. CT-AVC may also provide value in measuring disease progression, improving the progression-to-noise ratio previously discussed. Quantification of calcium by CT has been shown to demonstrate excellent interobserver reproducibility and scan-rescan repeatability; with limits of agreement approximately ±70 AU and variation of approximately 4% to 8%, respectively 37, 39, 41. Moreover, mean annual progression in calcium score is relatively large, ranging from approximately 141 AU/year in mild AS to 361 AU/year in severe AS. Whether CT calcium scoring will be modifiable with medical therapies is yet to be determined; however, its attributes have led to its adoption in the research setting as the primary efficacy endpoint in multiple ongoing studies investigating novel treatments for AS. CT calcium scoring is not currently recommended for routinely tracking disease progression in the clinical arena, although recent European Society of Cardiology guidelines support its use in adjudicating disease severity in patients with discordant echocardiographic assessments and normal flow (23).

## Positron Emission Tomography

**Figure 2:**
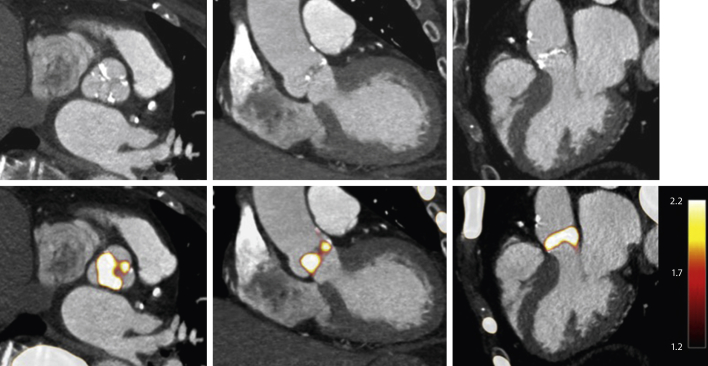
^18^F-NaF PET-CT of the Aortic Valve Contrast-enhanced computed tomography (CT) of the aortic valve **(top)** with fused radiolabeled sodium fluoride (^18^F-NaF) positron emission tomography (PET)–CT angiography images in the same patient **(bottom)**. Strong PET uptake can be localized to the aortic valve in short-axis **(left),** coronal **(middle),** and sagittal **(right)** views.

Positron emission tomography (PET) is another novel technique, the use of which is being explored in AS. By monitoring biological processes within the body, this modality has the potential to offer important mechanistic insights into pathophysiology. Furthermore, as a marker of disease activity and very early calcium formation, there is growing interest in using PET to detect early therapeutic effects in AS at a stage in the process when calcium is more likely to be reversible (Figure 2).

**Figure 3:**
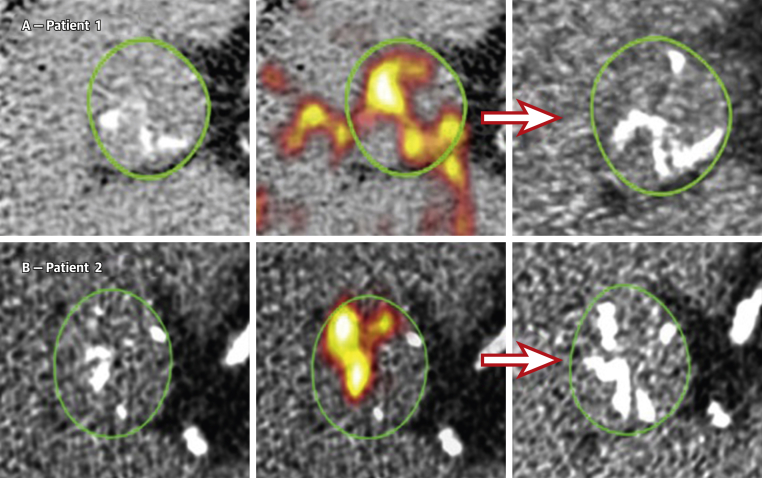
^18^F-NaF PET-CT Predicts Disease Progression in AS Baseline CT calcium score scans **(left)** for patients 1 **(A)** and 2 **(B).** Fused ^18^F-NAF PET-CT scans **(middle)** show fluoride uptake in **red** and **yellow**. Follow-up CT at 1 year **(right)** suggests that the baseline PET signal predicts where new macroscopic calcium, visible on the CT, is going to develop. Reproduced with permission from Dweck et al. (45). AS = aortic stenosis; other abbreviations as in Figures 1 and 2.

Radiolabeled sodium fluoride (^18^F-NaF) is a widely available PET tracer that can be used to measure calcification activity in the vasculature, with an affinity for developing microcalcification 42, 43, 44, 45. ^18^F-NaF activity is increased in patients with aortic valve disease compared with the healthy population, with a progressive rise in PET uptake with increasing severity of AS (jet velocity and calcium score) (39). Furthermore, early studies have suggested that ^18^F-NaF activity predicts the rate of future disease progression as measured by CT-AVC and echocardiography 45, 46. Indeed, new areas of macrocalcification appear to subsequently develop at sites of increased baseline ^18^F-NaF uptake, consistent with this tracer identifying developing calcification before it is visible on CT (Figure 3). As a marker of disease activity, ^18^F-NaF therefore holds potential in detecting therapeutic effects more rapidly than conventional anatomical imaging approaches do (47). Encouragingly, excellent reproducibility of this technique has been demonstrated (intraclass correlation coefficient [ICC]: >0.8), and scan-rescan reproducibility has also shown good agreement with a percentage error of ±10% (47). However, the incremental value of this modality beyond anatomical imaging modalities has yet to be shown and given the cost, availability of scanners, and radiation exposure, it is unlikely to be used clinically in the near future. However, ^18^F-NaF PET does hold promise in the research arena and as an endpoint in clinical trials of novel therapy, potentially being more sensitive to treatment effects than other imaging markers. Although it remains to be determined whether the aortic valve ^18^F-NaF PET signal is modifiable with medical therapy, the same is true for all imaging biomarkers in the absence of an effective medical therapy for this condition. Interestingly, skeletal ^18^F-NaF uptake in metabolic bone disease does appear to be modifiable, demonstrating clear changes after only 1 month of bisphosphonate therapy (48).

Another widely used PET tracer that has been explored in imaging of the aortic valve is fluorodeoxyglucose F 18. This glucose analogue becomes trapped in metabolically active cells and serves as a surrogate marker for macrophage burden and inflammation. In aortic stenosis, fluorodeoxyglucose F 18 activity is increased in patients with AS although in practice image interpretation is frequently challenging due to overspill of activity from the adjacent myocardium 39, 45. Finally, the development of PET–magnetic resonance imaging scanning platforms provides new opportunities to perform PET imaging studies at low radiation dose and potentially allowing multiple time points to be studied in individual patients (49).

## Assessing the Myocardium

In addition to monitoring progressive valve narrowing, noninvasive imaging may also provide detailed assessments of the LV remodeling response: from identifying initial compensatory hypertrophy to tracking subsequent decompensation with the development of progressive myocardial fibrosis and functional impairment. Such markers of LV decompensation (e.g., inappropriate increases in LV mass, high-sensitivity troponin levels, markers of myocardial fibrosis, global longitudinal strain, ejection fraction) are consistently associated with an adverse long-term prognosis in AS independent of disease severity, underlying the importance of myocardial health in this condition 50, 51, 52.

### Echocardiography

Echocardiography can provide assessments of LV mass and ejection fraction that are adequate for routine clinical use and are widely used for decision making. However, assessments of LV mass by echocardiography demonstrate poor reproducibility and scan-rescan repeatability with variability in measurements of between 20 and 40 g (29). Indeed, this variability far exceeds the rate of progression of LV mass (approximately 3 g/m^2^ per year), meaning that echocardiography is not well suited as an endpoint in research trials, mandating extremely large sample sizes and prolonged follow-up to detect even quite large treatment effects 29, 53.

Although echocardiography cannot directly assess myocardial fibrosis, surrogate markers provide insights into the functional consequences of this fibrosis. Ejection fraction is widely measured in clinical practice with reductions <50% an indication for aortic valve replacement. However, this measure only changes late in the disease process, also relies on geometric assumptions, and is often confounded in AS due to the presence of coexistent LV hypertrophy (29). Global longitudinal strain measures myocardial deformation by speckle tracking and, in asymptomatic patients with severe aortic stenosis, GLS may provide prognostic information while ejection fraction remains in the normal range 54, 55. Further work is required, however, to define relevant thresholds to aid decision making and the ability of GLS to track disease progression with time.

### Cardiac magnetic resonance

Cardiac magnetic resonance (CMR) imaging, compared with echocardiography, offers important advantages in the quantification of LV mass and ejection fraction. Excellent intra- and interobserver as well as interstudy repeatability have led to CMR becoming the gold standard for this purpose 56, 57, 58. Indeed, the interscan, interobserver, and intraobserver variability for CMR assessments of LV mass have each been documented as <10 g 56, 58. CMR therefore holds advantages in monitoring myocardial remodeling, potentially detecting even modest changes in response to therapy. This was highlighted by the RIAS (Ramipril in Aortic Stenosis) trial, which enrolled 100 patients with AS and demonstrated a subtle but statistically significant reduction in LV mass after 1 year in patients treated with ramipril (−4 g) versus a similar increase in LV mass (+4.5 g) in the placebo group (59).

**Figure 4:**
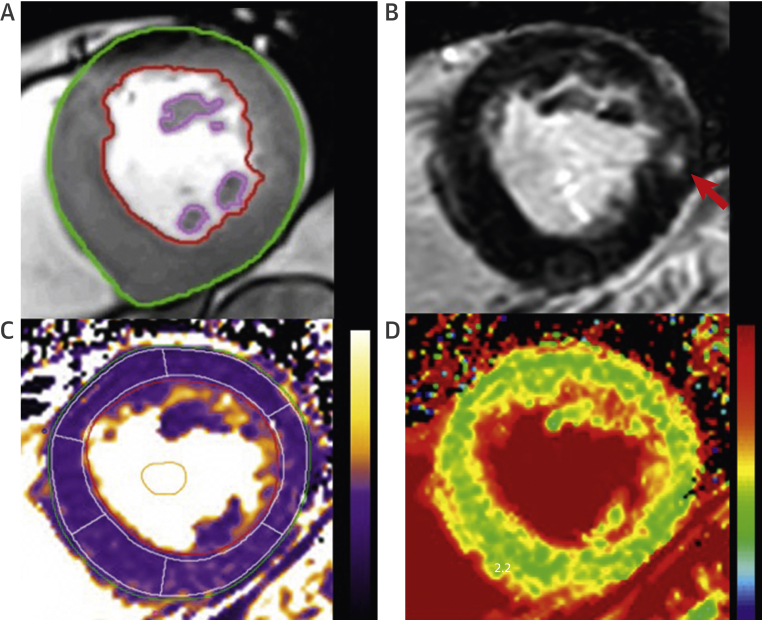
Myocardial Mass Quantification and Tissue Characterization by CMR Endocardial and epicardial contours can be accurately contoured to provide quantification of left ventricular mass **(A)**. Midwall fibrosis on delayed enhancement imaging **(red arrow) (B)**. Native T_1_ map **(C)** and extracellular volume fraction map **(D)**. CMR = cardiac magnetic resonance.

Another major advantage of CMR is its ability to provide noninvasive myocardial tissue characterization using late gadolinium enhancement (LGE) and T_1_ mapping techniques (Figure 4). Gadolinium has been widely used as an extracellular CMR contrast agent, potently shortening tissue T_1_ values following intravenous administration. Altered kinetics in areas of extracellular expansion can be exploited using inversion recovery LGE sequences. By manually setting the inversion time to “null” normal myocardium, these areas appear black. Conversely, areas of replacement fibrosis are characterized by accumulation and delayed washout of gadolinium, thereby appearing white and facilitating the visualization of myocardial scarring. In AS, replacement fibrosis occurs in a characteristic mid-wall pattern and can be detected in 29% to 62% of patients 52, 60, 61. Replacement fibrosis may be quantified using semiautomated techniques that are reproducible between observers (ICC: >0.9; coefficient of reproducibility: 16 mm^2^) 62, 63. Once present, replacement fibrosis appears to accumulate rapidly, increasing by up to 78% per year (53), and both its presence and increasing burden are strong predictors of adverse outcome 52, 61, 64. Although progression may be halted by aortic valve intervention, once present, replacement fibrosis appears to be irreversible 53, 64, 65. The presence of mid-wall LGE has therefore been suggested as an objective marker of LV decompensation that can be used to optimize the timing of valve intervention. Indeed this strategy is currently being tested in the ongoing EVOLVED (Early Valve Replacement Guided by Biomarkers of LV Decompensation in Asymptomatic Patients With Severe AS) randomized controlled trial (NCT03094143).

Myocardial fibrosis may also be present in a diffuse interstitial pattern, which is not detected by LGE techniques. Instead, this process may be identified and quantified using T_1_ mapping techniques that estimate absolute T_1_ values in a voxel-by-voxel map. Native T_1_ values are raised in patients with severe AS compared with healthy volunteers (66) and provide prognostic information (67). However, there is significant overlap in native T_1_ values between patients and healthy volunteers, between different sequences, and between different magnetic field strengths, which have thus far limited its widespread application. Whereas reproducibility of native T_1_ values is excellent between observers (ICC: 0.99), repeatability is more variable with an ICC of 0.72 for scan-rescan measurements (68).

**Figure 5:**
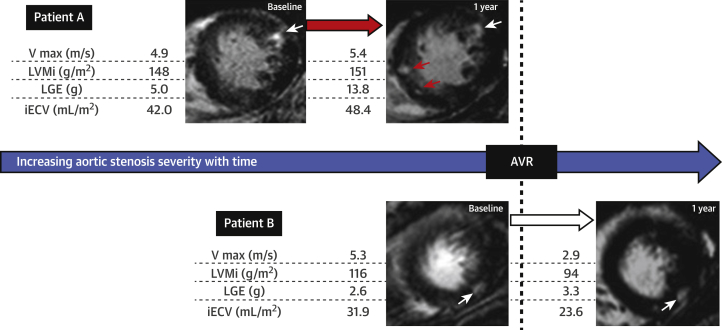
Changes in LVMi, Diffuse Fibrosis (iECV), and Replacement Fibrosis (LGE) in AS Before and After AVR In Patient A, new areas of late gadolinium enhancement (LGE) are seen on follow-up **(red arrow).** In Patient B, following aortic valve replacement (AVR), both cellular hypertrophy and the total myocardial fibrosis burden (iECV) regress. By comparison, replacement fibrosis appear irreversible **(white arrow)**. Reproduced with permission from Everett et al. (53). AS = aortic stenosis; iECV = indexed extracellular volume; LVMi = left ventricular mass index; Vmax = peak aortic-jet velocity.

T_1_ mapping can also be repeated following administration of gadolinium, which enables calculation of the extracellular volume fraction (ECV%) (i.e., the fraction of the myocardial volume that is extracellular space). This measure has been well validated against collagen volume fraction on histology 69, 70, 71 as a surrogate of diffuse fibrosis and can also discriminate between healthy volunteers and patients (68), although again with significant overlap. Measurements of ECV fraction have been found to be highly reproducible between observers and serial scans (ICC: 0.97 and 0.96, respectively) 68, 70. An advantage is that ECV fraction removes much of the variation attributed to sequence and magnetic field strength, so values are potentially comparable between centers. However, more recent data have demonstrated that balanced increases in both cellular hypertrophy and ECV occur as the remodeling response progresses, and that the ECV fraction appears to increase, not decrease as one might expect, following AVR (due to more rapid myocyte than fibrosis regression). ECV fraction would therefore appear to have limitations as a method of tracking myocardial fibrosis burden with time or in tracking response to therapy (53). The indexed ECV (iECV) is an alternative yet related parameter that acts as a surrogate of the total myocardial fibrosis burden. It is calculated by multiplying the ECV fraction by the indexed volume of the myocardium and may better reflect temporal changes in the total burden of diffuse fibrosis (51) (Figure 5). In addition, indexed ECV appears clearly reversible following valve replacement 53, 72 and may offer improved differentiation between disease states. Indexed ECV is therefore potentially the most attractive measure for assessing myocardial fibrosis progression in AS, with further multicenter trials required for validation.

## Use of Imaging to Test Drug Efficacy

As yet, no medical therapy has proven effective in reducing progression of AS or improving clinical outcomes in patients with this condition. However, as our understanding of the complex pathophysiological processes underlying AS improves, novel therapeutic strategies have been developed and are under active investigation. A challenge is to identify patients in whom the progressive cycle of calcification may be reversible with effective therapy, and this may be more likely to occur early in stages of calcium formation. Most randomized trials to date have targeted patients with mild or moderate AS, and it has been suggested that patients would be more effectively treated at an earlier stage. However, as the majority of patients with aortic sclerosis do not develop AS, identifying patients at an earlier stage who are likely to derive benefit from treatment would be challenging. Furthermore, many patients do not present until they have developed advanced disease and so developing therapies that halt disease progression in this patient group is also of great importance. Regardless of the stage of disease, it appears probable that calcification is more likely to be reversible in its earlier stages of development, meaning that imaging techniques that can identify early developing calcification, such as ^18^F-NaF PET, are likely to be of value (Table 1).

**Table 1:** Current Trials Investigating the Effects of Novel Therapies on Progression of AS

Trial Name	NCT #	Therapy	Population	Imaging Endpoints	Primary Outcome
EAVall (Early Aortic Valve Lipoprotein(a) Lowering)	NCT02109614	Niacin vs. placebo	238 participants with aortic sclerosis or mild AS	CT calcium score Echo	Change in CT calcium score at 2 yrs
SALTIRE-II (Study Investigating the Effect of Drugs Used to Treat Osteoporosis on the Progression of Calcific Aortic Stenosis)	NCT02132026	Alendronate or denosumab vs. placebo	150 patients with AV Vmax >2.5 m/s and grade 2–4 calcification on echo	CT calcium score Echo ^18^F-NaF PET-CT	Change in CT calcium score at 2 yrs
PCSK9 Inhibitors in the Progression of Aortic Stenosis	NCT03051360	PCSK9 inhibitor vs. placebo	140 patients with mild to moderate AS	CT calcium score Echo ^18^F-NaF PET-CT	Change in calcium score and ^18^F-NaF PET activity at 2 yrs
BASIK2 (Bicuspid Aortic Valve Stenosis and the Effect of Vitamin K2 on Calcium Metabolism on 18F-NaF PET/MRI)	NCT02917525	Vitamin K2 vs. placebo	44 patients with a bicuspid AV and mild to moderate calcification on echo	CT calcium score Echo ^18^F-NaF PET-MRI	Change in ^18^F-NaF PET activity at 6 months
CALCIFICA (Value of Oral Phytate [InsP6] in the Prevention of Progression of Cardiovascular Calcifications)	NCT01000233	Phytate vs. placebo	250 patients with grade 2 or 3 AV calcification on echo	CT calcium score	CT calcium score at 2 yrs
ASPEN (Aortic Stenosis and Phosphodiesterase Type 5 Inhibition)	NCT01275339	Tadalafil vs. placebo	With moderate to severe AS (AVA <1.5 cm)	MRI Echo	Change in LV mass at 6 months Change in diastolic function on echo Change in LV longitudinal strain on echo
CAVS (A Study Evaluating the Effects of Ataciguat [HMR1766] on Aortic Valve Calcification)	NCT02481258	Ataciguat (HMR1766) vs. placebo	35 patients with AVA between 1 and 2 cm^2^ and calcium score >300 AU + EF >50%.	CT calcium score Echo	Change in CT calcium score at 6 and 12 months

A summary of current trials employing imaging endpoints to assess the effects of novel therapies by utilizing imaging endpoints.

AS = aortic stenosis; AU = arbitrary unit(s); AV = aortic valve; AVA = aortic valve area; CT = computed tomography; echo = echocardiography; EF = ejection fraction; ^18^F-NaF = radiolabeled sodium fluoride; LV = left ventricular; MRI = magnetic resonance imaging; NCT = national clinical trial; PCSK9 = proprotein convertase subtilisin/kexin type 9; PET = positron emission tomography; Vmax = maximum volume.

### Lipid-lowering strategies

Although statin therapy has failed to slow disease progression in 3 randomized trials 73, 74, 75, the question remains as to whether alternative lipid-lowering approaches could be successful. One promising therapeutic target is lipoprotein(a) [Lp(a)], a cholesterol-rich plasma lipoprotein containing a low-density lipoprotein particle with apolipoprotein B100 bound to apolipoprotein A (76). Lp(a) has been recognized as a powerful cardiovascular risk factor (76), and a recent genome-wide association study has implicated a single-nucleotide polymorphism in the Lp(a) locus to the development of the disease (77).

The growing evidence that Lp(a) plays a causal role in the development and also perhaps the progression of AS 78, 79 has led to great enthusiasm in exploring this as a therapeutic target. One potential therapeutic agent is niacin, which has been shown to lower Lp(a) in a dose-dependent manner. Whether extended-release niacin can reduce the progression of aortic valve disease is currently under investigation as part of the EAVall (Early Aortic Valve Lipoprotein(a) Lowering Trial) (NCT02109614). This pilot study will randomize over 200 patients with elevated Lp(a) and mild aortic valve disease to extended-release niacin or placebo with a primary outcome of change in CT-AVC at 2 years (79).

### Anticalcific therapies

Calcification is a key target for novel therapies in AS given its central role in driving progressive valvular obstruction. An important concern, however, is how best to reduce calcification activity in the valve while maintaining bone health in elderly patients with AS who are at risk of osteoporosis and fractures. One potential option is to use treatments licensed for osteoporosis, such as bisphosphonates or receptor activator of nuclear factor kappa-B ligand (RANKL)-inhibitors, which not only improve bone health but have also demonstrated the ability to reduce vascular calcification (80). In addition to reducing bone resorption and thereby reducing circulating calcium and phosphate, bisphosphonates have been shown to reduce local production of inflammatory cytokines, inhibit release of matrix metalloproteinases, and stimulate macrophage apoptosis 81, 82. Preclinical studies have demonstrated that bisphosphonate therapy reduces valvular calcification in animal models 83, 84. The monoclonal antibody to RANKL, denosumab, acts by preventing the interaction between RANKL and RANK, a cytokine that plays an important role in up-regulating pro-osteogenic mediators and inducing osteoblastic transformation of valve interstitial cells. Whether these therapies reduce the progression of valvular calcification has yet to be determined and is currently being investigated in the randomized placebo-controlled trial SALTIRE II (Study Investigating the Effects of Drugs Used to Treat Osteoporosis on the Progression of Calcific Aortic Stenosis) (NCT02132026). This blinded study is using change in CT calcium score at 2 years as the primary efficacy endpoint. However, measures of calcification activity by PET-CT will also be performed at baseline and 1 year, in addition to serial echocardiography every 6 months (85).

Another potential anticalcific therapy under investigation is vitamin K. Vitamin K is required for activation of matrix Gla protein (MGP), a potent inhibitor of vascular calcification synthesized by vascular smooth muscle cells. In the aortic valve, MGP acts by blocking the binding of bone morphogenetic protein 2 to its receptor, thereby preventing bone morphogenetic protein–mediated differentiation of valve interstitial cells to pro-osteogenic cells. MGP also inhibits growth of microcalcification crystals by binding directly to hydroxyapatite and stabilizing circulating calcifying protein particles (86). Reduced expression of MGP has been demonstrated in calcific aortic valves (87). The BASIK2 (Bicuspid Aortic Valve Stenosis and the Effect of Vitamin K2 on Calcium Metabolism on 18F-NaF PET/MRI) randomized trial (NCT02917525) will investigate the effect of vitamin K2 in 44 patients with bicuspid aortic valve disease. The primary outcome of the study is the change in ^18^F-fluoride PET signal on PET-MRI at 6 months (86).

### Antifibrotic strategies

Therapies targeting the remodeling response of the LV have also been of interest. Whereas the RIAS trial of angiotensin-converting enzyme–inhibitor therapy showed a small positive effect on LV mass, a small and underpowered study did not demonstrate an effect of eplerenone on LV mass progression (88). Further work in this field is required particularly to investigate the effects of novel therapies on myocardial fibrosis (59), with hope that more aggressive inhibition of the renin-angiotensin-aldosterone system and or novel antifibrotic therapies may prove more effective 89, 90, 91.

## Conclusions

AS represents a substantial health care burden, with no medical therapies currently available to intervene and halt disease progression in either the valve or myocardium. However, advances in our understanding of the complex pathophysiological basis of this disease have led to exciting new avenues in exploring potential therapies. Investigating the effects of new treatments requires objective markers that are both repeatable and sensitive to changes. Although echocardiographic measures are used widely to guide clinical practice, alternative imaging modalities such as CT calcium scoring, CMR, and 18F-fluoride PET imaging are being used to assess disease activity and progression in the research arena and as efficacy endpoints in ongoing trials of novel therapies.
